# The methylated N-terminal tail of RCC1 is required for stabilisation of its interaction with chromatin by Ran in live cells

**DOI:** 10.1186/1471-2121-11-43

**Published:** 2010-06-21

**Authors:** Ekarat Hitakomate, Fiona E Hood, Helen S Sanderson, Paul R Clarke

**Affiliations:** 1 Biomedical Research Institute, School of Medicine, College of Medicine, Dentistry and Nursing, University of Dundee, Ninewells Hospital and Medical School, Dundee DD1 9SY, UK; 2 The Physiological Laboratory, School of Biomedical Sciences, University of Liverpool, Liverpool L69 3BX, UK

## Abstract

**Background:**

Regulator of chromosome condensation 1 (RCC1) is the guanine nucleotide exchange factor for Ran GTPase. Localised generation of Ran-GTP by RCC1 on chromatin is critical for nucleocytoplasmic transport, mitotic spindle assembly and nuclear envelope formation. Both the N-terminal tail of RCC1 and its association with Ran are important for its interaction with chromatin in cells. In vitro, the association of Ran with RCC1 induces a conformational change in the N-terminal tail that promotes its interaction with DNA.

**Results:**

We have investigated the mechanism of the dynamic interaction of the α isoform of human RCC1 (RCC1α) with chromatin in live cells using fluorescence recovery after photobleaching (FRAP) of green fluorescent protein (GFP) fusions. We show that the N-terminal tail stabilises the interaction of RCC1α with chromatin and this function can be partially replaced by another lysine-rich nuclear localisation signal. Removal of the tail prevents the interaction of RCC1α with chromatin from being stabilised by Ran^T24N^, a mutant that binds stably to RCC1α. The interaction of RCC1α with chromatin is destabilised by mutation of lysine 4 (K4Q), which abolishes α-N-terminal methylation, and this interaction is no longer stabilised by Ran^T24N^. However, α-N-terminal methylation of RCC1α is not regulated by the binding of Ran^T24N^. Conversely, the association of Ran with precipitated RCC1α does not require the N-terminal tail of RCC1α or its methylation. The mobility of RCC1α on chromatin is increased by mutation of aspartate 182 (D182A), which inhibits guanine-nucleotide exchange activity, but RCC1α^D182A ^can still bind nucleotide-free Ran and its interaction with chromatin is stabilised by Ran^T24N^.

**Conclusions:**

These results show that the stabilisation of the dynamic interaction of RCC1α with chromatin by Ran in live cells requires the N-terminal tail of RCC1α. α-N-methylation is not regulated by formation of the binary complex with Ran, but it promotes chromatin binding through the tail. This work supports a model in which the association of RCC1α with chromatin is promoted by a conformational change in the α-N-terminal methylated tail that is induced allosterically in the binary complex with Ran.

## Background

The small Ran GTPase plays key roles during the cell cycle in eukaryotic cells [1]. Generation of RanGTP from RanGDP requires a Ran guanine nucleotide exchange factor (RanGEF) known as Regulator of Chromosome Condensation 1 (RCC1) in vertebrates [2,3]. RCC1 is localised predominantly to chromatin throughout the cell cycle [4,5]. Hydrolysis of GTP to GDP by Ran is greatly stimulated by Ran GTPase-activating protein (RanGAP) in the cytoplasm [6]. The distinct localisation of these regulators results in a high concentration of RanGTP relative to that of RanGDP in the vicinity of chromatin [7]. Within the nucleus, RanGTP promotes the assembly of export complexes between proteins carrying a leucine-rich nuclear export signal (NES) and exportin (Crm1), while causing the disassembly of imported complexes formed between proteins carrying a lysine-rich nuclear import signal (NLS) and importins. Thus, RanGTP determines the direction of nucleocytoplasmic transport during interphase [8]. In animal cells in which the nuclear envelope breaks down during mitosis and the separation of the nucleoplasm and cytoplasm is lost, continued generation of RanGTP on chromosomes by RCC1 is thought to provide a spatial signal to organise spindle assembly [9]. Localised generation of RanGTP by RCC1 on chromatin is therefore critical for the function of the Ran system throughout the cell cycle [1].

RCC1 has a core domain with a 7-bladed propeller structure [10] that interacts on one face with Ran [11] and is proposed to interact on the other face with chromatin [12,13], possibly through core histones H2A and H2B [14]. Near to the N-terminus is a short flexible region that contains a functional lysine-rich nuclear localisation signal (NLS) that associates with the import receptor dimer formed by importin-α3 and importin-β [5,15,16]. In vitro, this basic N-terminal region (NTR) or tail can interact directly with DNA [13,17] and in cells it is involved in both the concentration of RCC1 in the nucleus and in its interaction with chromatin [5]. RCC1 is modified in cells by removal of the initial N-terminal methionine and mono-, di- or tri-methylation of the α-amino group of the new N-terminal residue (serine 2 in human RCC1). This modification is present throughout the cell cycle and promotes the localisation of RCC1 to mitotic chromosomes [18]. During mitosis, phosphorylation of RCC1 at serine 2 and serine 11 by CDK1-cyclin B1 dissociates RCC1 from importin-α3-importin-β and regulates its interaction with chromatin [19,20]. In mammalian cells, RCC1 exists in at least three isoforms (α, β and γ), which are probably generated by alternative splicing of the mRNA. RCC1β and RCC1γ have unique inserts after residue 24 which alter the length of their N-terminal tails. In the case of RCC1γ, a 17 amino acid insert stabilises its interaction with chromatin, reduces importin binding and alters its regulation by phosphorylation at serine 11 [21].

Studies using RCC1 fused to green fluorescent protein (GFP) have shown that its interaction with chromatin in live cells is highly dynamic [19,22,23]. The rate of fluorescence recovery after photobleaching (FRAP) on chromatin is regulated by the association of GFP-RCC1α with Ran [19,22]. Mutation of aspartate 182 of RCC1α (D182A), which inhibits its guanine nucleotide exchange activity, destabilises the interaction of its GFP fusion with chromatin [5,19]. Conversely, co-expression of Ran^T24N^, a mutant defective in nucleotide binding that forms a stable complex with RCC1 and inhibits its guanine nucleotide exchange activity [24,25], strongly stabilises the interaction of GFP-RCC1α with chromatin [22]. GFP-Ran^T24N ^associates stably with chromatin throughout the cell cycle and co-localises with RCC1, consistent with formation of a stable binary complex with RCC1 [26]. Li et al. [22] proposed that the association of RCC1 with nucleotide-free Ran (apoRan) in a transient binary complex stabilises its interaction with chromatin, thereby linking the interaction of RCC1 with chromatin to generation of Ran-GTP. More recently, Hao and Macara [13] have developed a fluorescence resonance energy transfer (FRET)-based reporter in which RCC1α was fused to CFP at its N-terminal and YFP at its C-terminal (CFP-RCC1α-YFP). They showed that binding of Ran^T24N ^or apoRan^WT ^to CFP-RCC1α-YFP caused a conformational change in the N-terminal tail, which stabilised the interaction of CFP-RCC1α-YFP with DNA in vitro and with chromatin in permeabilised cells. These authors proposed a model in which the interaction of Ran with RCC1 allosterically induces a conformational change in the N-terminal tail, which then interacts with DNA and thereby stabilises the association of RCC1 with chromatin. In these experiments, however, the role of α-N-methylation, which was by necessity abolished in the CFP-RCC1α-YFP reporter, could not be tested. The role of the N-terminal tail in the interaction of the RCC1-Ran binary complex with chromatin also remained to be demonstrated in vivo.

Here, we have tested the mechanism of the interaction of RCC1α with chromatin in live cells using FRAP of GFP fusions. We show that the α-N-methylated tail of RCC1α is important for the stability of the interaction of RCC1α with interphase chromatin. Indeed, the methylated tail is required for the stabilising effect of Ran^T24N^. These results provide strong support for an allosteric model of the interaction of the RCC1-Ran binary complex with chromatin in vivo.

## Results

### The N-terminal tail of RCC1 is required for stable association with interphase chromatin

**Figure 1:**
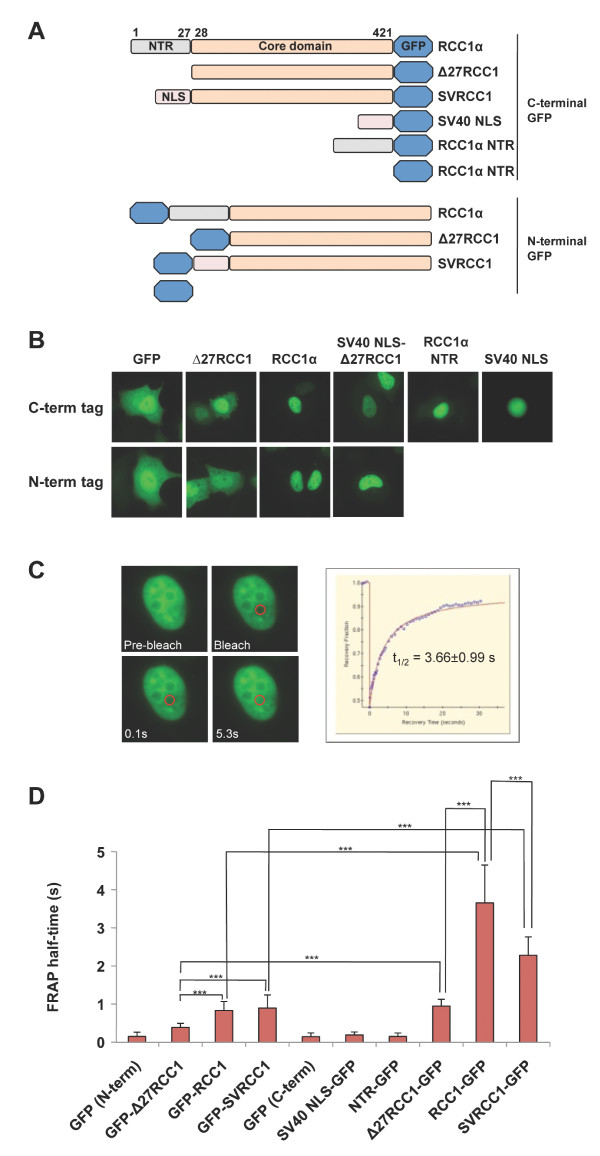
**The N-terminal region (NTR) or tail of RCC1α is required for stable interaction with chromatin in live cells**. (A) Schematic diagrams of N-terminal and C-terminal GFP fusion constructs. (B) Representative images of live HeLa cells expressing the N-terminal and C-terminal GFP fusion constructs. (C) Fluorescence recovery after photobleaching (FRAP) of RCC1α-GFP. Images of a cell during the timecourse are shown (left). Circles indicate the region of photobleaching. Output of data (right) with FRAP half-time (t_1/2_). (D) Mean of the FRAP t_1/2 _for each GFP fusion construct. Data from 3 independent experiments were combined and tested for significant difference in FRAP t_1/2 _using the one-way ANOVA test. P-value less than 0.05 was considered to be statistically different (p-value < 0.001 shows as ***).

To examine the role of the N-terminal region (NTR) or tail of RCC1α in its dynamic interaction with chromatin in live cells, we made N-terminal and C-terminal GFP fusion constructs of the RCC1α N-terminal tail (residues 1-27), the RCC1 core domain (Δ27RCC1) and full-length RCC1α. We also fused the RCC1 core domain with a classical lysine-rich monopartite nuclear localisation signal (PKKKRK) derived from SV40 T antigen (SV40 NLS) to generate SV40 NLS-Δ27RCC1 (Figure 1A). When expressed in HeLa cells, this GFP fusion construct was concentrated in nuclei like full length RCC1α (Figure 1B). GFP proteins expressed from the same vectors as the N-terminal and C-terminal tagged proteins (N-term-GFP and C-term-GFP, respectively) were used as controls. To monitor the dynamic interaction between RCC1 with chromatin, we performed fluorescent recovery after photobleaching (FRAP) experiments on nuclei in live HeLa cells expressing the GFP fusion constructs (Figure 1C).

As expected, N-term-GFP alone freely diffused in nuclei with half-time (t_1/2_) of 0.15 ± 0.11 s (n = 33) (Figure 1D) (See Additional file 1 Figure S1 for original data). The FRAP half-time of GFP-RCC1α was 0.84 ± 0.23 s (n = 114) whereas t_1/2 _of GFP-Δ27RCC1 was decreased to 0.39 ± 0.11 s (n = 48), consistent with a stabilising role for the N-terminal tail in the interaction of RCC1α with interphase chromatin [19]. However, GFP-SV40 NLS-Δ27RCC1 was as mobile as the full-length RCC1α with the FRAP t_1/2 _of 0.90 ± 0.34 s (n = 96). These data indicate that, in the context of the N-terminal fusion with GFP, which cannot be α-N-methylated, the positively charged residues in the NLS of RCC1α weakly stabilise its interaction with chromatin. When the GFP is fused to the N-terminus, the weak stabilising effect is not specific to the tail of RCC1α and it can be replaced by another lysine-rich NLS-containing sequence.

When full-length RCC1α fused to GFP at its C-terminus was monitored it exhibited a much more stable association with chromatin than the N-terminal GFP tagged fusion protein, with a FRAP t_1/2 _of 3.66 ± 0.99 s (n = 79) (Figure 1D). Δ27RCC1-GFP was considerably less stable (0.95 ± 0.18 s., n = 23) than RCC1α-GFP, but was still significantly more stable than GFP-Δ27RCC1. The FRAP t_1/2 _of SV40 NLS-Δ27RCC1-GFP was 2.29 ± 0.48 s. (n = 29), which was intermediate between Δ27RCC1-GFP and RCC1α-GFP. Thus, the free N-terminal tail of RCC1α stabilises the association of protein with interphase chromatin in live cells. This stabilising effect can be partially reproduced by another basic NLS sequence.

To test whether the N-terminal tail alone is sufficient to interact with chromatin, the mobility of the isolated NTR-GFP was monitored. The FRAP t_1/2 _of the isolated NTR-GFP (0.15 ± 0.09 s, n = 82) was as mobile as that of the GFP alone (0.15 ± 0.10 s, n = 54). Similarly, the monopartite SV40 NLS-GFP was nuclear and the FRAP t_1/2 _(0.19 ± 0.08 s, n = 60) was not different from either GFP alone or NTR-GFP. Thus, the NLS-containing N-terminal tail of RCC1α is insufficient to stabilise interaction with chromatin unless it is joined to the core domain.

### Stable Ran binding to RCC1 regulates the dynamic interaction of RCC1 with chromatin in an N-terminal tail-dependent manner in live cells

**Figure 2:**
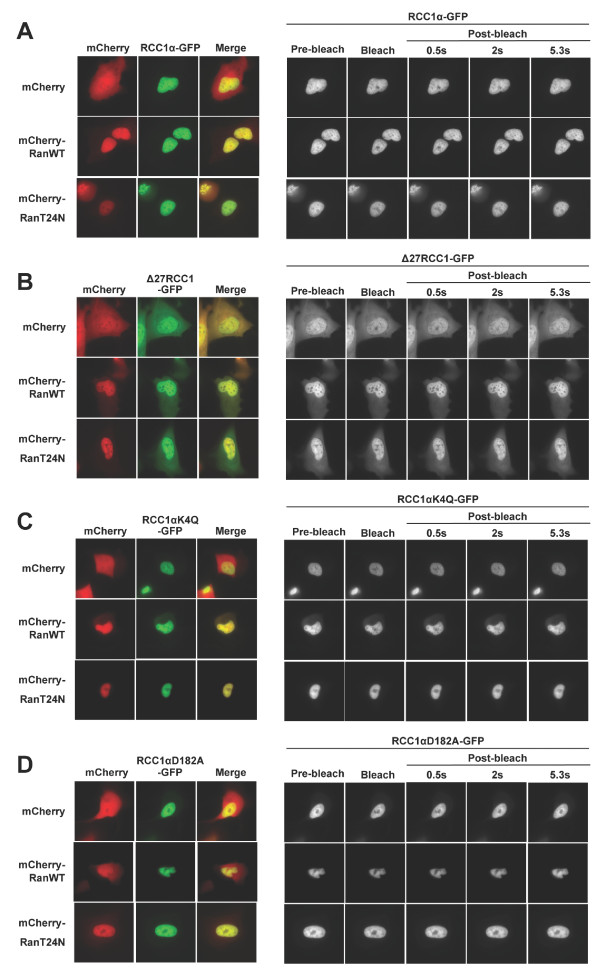
**Effect of Ran on the interaction of RCC1α with chromatin in live cells**. Representative images of live U2OS cells co-expressing wild-type RCC1α-GFP (A), Δ27RCC1-GFP (B), RCC1α^K4Q^-GFP (C) or RCC1α^D182A^-GFP (D) with mCherry empty vector (top), mCherry-Ran^WT ^(middle) or mCherry-Ran^T24N ^(bottom) during fluorescence recovery after photobleaching (FRAP).

**Figure 3:**
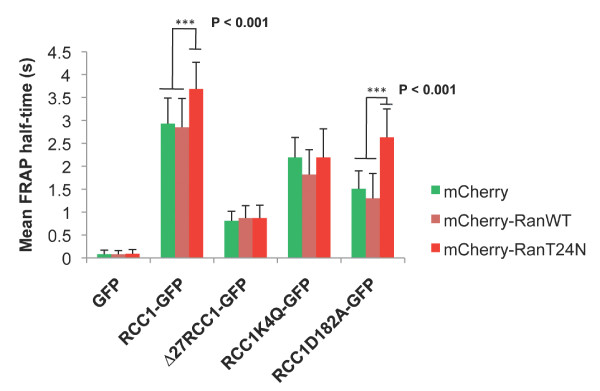
**Stabilisation of the interaction of RCC1α with chromatin by Ran requires the methylated N-terminal tail of RCC1α**. Bar chart showing the mean FRAP t_1/2 _+/- SD of live U2OS cells co-expressing wild-type RCC1α-GFP, Δ27RCC1-GFP, RCC1α^K4Q^-GFP or RCC1α^D182A^-GFP with mCherry empty vector (green), mCherry-Ran^WT ^(pink) or mCherry-Ran^T24N ^(red). Data were tested for significant difference in FRAP t_1/2 _using the Student's *t*-test. P-values considered to be statistically different (< 0.001) are indicated as ***.

We next confirmed that the binding of Ran^T24N ^to RCC1α stabilises the interaction of RCC1α with chromatin in live human cells. U2OS cells were transiently co-transfected with GFP alone or RCC1α-GFP together with the fluorescent protein mCherry alone, mCherry fused to wild-type Ran (mCherry-Ran^WT^) or mCherry fused to Ran^T24N ^(mCherry-Ran^T24N^). As expected, the FRAP t_1/2 _of RCC1α-GFP in cells co-expressing mCherry-Ran^T24N ^was significantly increased compared to that of RCC1α-GFP in cells co-expressing either mCherry or mCherry-Ran^WT ^(Figure 2A, Figure 3, Table 1). In addition, the mobile fraction of RCC1α-GFP, calculated as the proportion of the initial fluorescent signal that is recovered after photobleaching, was significantly reduced in cells in which mCherry-Ran^T24N ^was co-expressed with RCC1α-GFP compared to cells co-expressing either mCherry or mCherry-Ran^WT ^(Table 1). Therefore, Ran^T24N ^specifically stabilises the interaction of RCC1α with chromatin in live cells.

**Table 1:** Stable binding of Ran with RCC1 regulates chromatin binding of RCC1 through the N-terminal tail in live cells.

Constructs	Mean FRAP half-time ± SD (s)	Mean mobile fraction ± SD	n
GFP + mCherry	0.08 ± 0.09	0.92 ± 0.06	19
GFP + mCherry-RanWT	0.08 ± 0.08	0.89 ± 0.04	14
GFP + mCherry-RanT24N	0.09 ± 0.09	0.91 ± 0.06	17

RCC1α-GFP + mCherry	2.93 ± 0.56	0.91 ± 0.05	21
RCC1α-GFP + mCherry-RanWT	2.85 ± 0.63	0.91 ± 0.04	26
RCC1α-GFP + mCherry-RanT24N	3.69 ± 0.58	0.87 ± 0.05	21

Δ27RCC1-GFP + mCherry	0.81 ± 0.21	0.94 ± 0.05	23
Δ27RCC1-GFP + mCherry-RanWT	0.87 ± 0.27	0.95 ± 0.04	23
Δ27RCC1-GFP + mCherry-RanT24N	0.87 ± 0.28	0.94 ± 0.04	26

RCC1αK4Q-GFP + mCherry	2.19 ± 0.44	0.90 ± 0.06	27
RCC1αK4Q-GFP + mCherry-RanWT	1.82 ± 0.54	0.90 ± 0.06	26
RCC1αK4Q-GFP + mCherry-RanT24N	2.19 ± 0.63	0.90 ± 0.07	30

RCC1αD182A-GFP + mCherry	1.51 ± 0.39	0.93 ± 0.04	20
RCC1αD182A-GFP + mCherry-RanWT	1.30 ± 0.54	0.94 ± 0.05	10
RCC1αD182A-GFP + mCherry-RanT24N	2.63 ± 0.62	0.87 ± 0.05	12

Fluorescence recovery after photobleaching (FRAP) data derived from live U2OS cells co-expressing GFP, RCC1α-GFP, Δ27RCC1-GFP, RCC1α K4Q-GFP, RCC1αD182A. The table summarises the mean +/- standard deviation (SD) of FRAP half-time and mobile fraction of each of the GFP fusion constructs in the nuclei of cells that also expressed mCherry, mCherry-RanWT or mCherry-RanT24N. The number of cells used in each case is given (n).

To test whether stabilization of interaction of RCC1α with chromatin by Ran^T24N ^requires the N-terminal tail of RCC1α, we co-expressed a series of RCC1α-GFP fusion proteins (C-term GFP tagged) with mCherry alone, mCherry-Ran^WT ^and mCherry-Ran^T24N ^in U2OS cells. As expected, RCC1α-GFP localized to the nucleus, as did RCC1α^K4Q^-GFP and RCC1α^D182A^-GFP, whereas Δ27RCC1-GFP was also present in the cytoplasm (Figure 2, left panels). FRAP experiments on these GFP fusions (Figure 2, right panels) showed that the FRAP t_1/2 _of RCC1α-GFP was significantly increased when mCherry-Ran^T24N ^was co-expressed compared to when mCherry or mCherry-Ran^WT ^were co-expressed (Table 1; Figure 3). The truncated version of RCC1 lacking the N-terminal tail (Δ27RCC1-GFP) showed a dramatic decrease in the FRAP t_1/2 _(0.81 ± 0.21 s.) compared to the full-length protein (RCC1α-GFP) (2.93 ± 0.56 s). However, Δ27RCC1-GFP failed to interact more stably in the presence of mCherry-Ran^T24N ^(0.87 ± 0.28 s). These results show that the binding of Ran to RCC1α stabilises the interaction of RCC1α with chromatin in an N-terminal tail-dependent manner in live cells. Consistent results were also found using N-terminal GFP fusions of RCC1α, although the proteins were all more dynamic (less stable) in their interactions with chromatin than the equivalent C-terminal GFP tagged proteins (Additional file 2 Figure S2).

### α-N-terminal methylation of RCC1α is required for the stabilization of its interaction with chromatin by Ran^T24N^

RCC1α is α-N-terminally methylated throughout the cell cycle and this modification promotes the localisation of RCC1α to mitotic chromosomes [18]. To test whether α-N-terminal methylation is important for dynamic association of RCC1 with interphase chromatin, we made a mutant of RCC1α (K4Q), which prevents methylation [18]. As expected, α-N-terminal methylation was completely blocked in RCC1α^K4Q^-GFP and was also absent in Δ27RCC1-GFP but was present in RCC1α-GFP, as well as RCC1α^D182A^-GFP, which has inhibited guanine nucleotide exchange activity [12] (Additional file 3 Figure S3). The mobility of RCC1α^K4Q^-GFP on interphase chromatin was significantly increased (t_1/2 _decreased) compared to that of wild-type RCC1α-GFP (Table 1, Figure 3) (p < 0.001). RCC1α^K4Q^-GFP also did not associate with chromatin more stably in cells co-expressing Ran^T24N^, in contrast to wild type RCC1α-GFP. Thus, α-N-terminal methylation of RCC1α plays a role in its dynamic interaction with interphase chromatin and this modification is important for stabilisation of the interaction with chromatin by Ran^T24N^.

### α-N-terminal methylation of RCC1α is not regulated by Ran^T24N^

**Figure 4:**
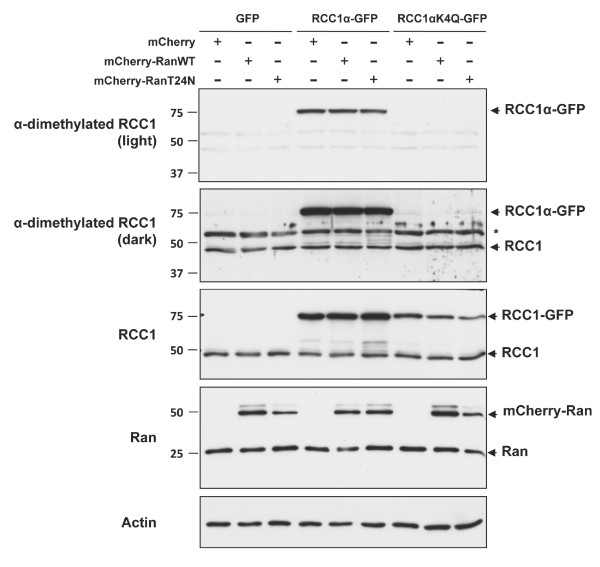
**α-N-terminal methylation of RCC1α is not affected by the stable association of Ran**. U2OS cells were co-transfected with GFP, RCC1α-GFP or RCC1α^K4Q^-GFP and mCherry, mCherry-Ran^WT ^or mCherry-Ran^T24N^. Cell lysates were analysed by Western blotting for α-dimethylated RCC1, RCC1, Ran and actin as a loading control. Methylated RCC1α-GFP was revealed on a light exposure of the α-dimethylated RCC1 blot and endogenous RCC1 isoforms on a dark exposure. A non-specific reactive band noted by [18] is indicated by *. The migration position of molecular mass markers (kDa) are shown left.

**Figure 5:**
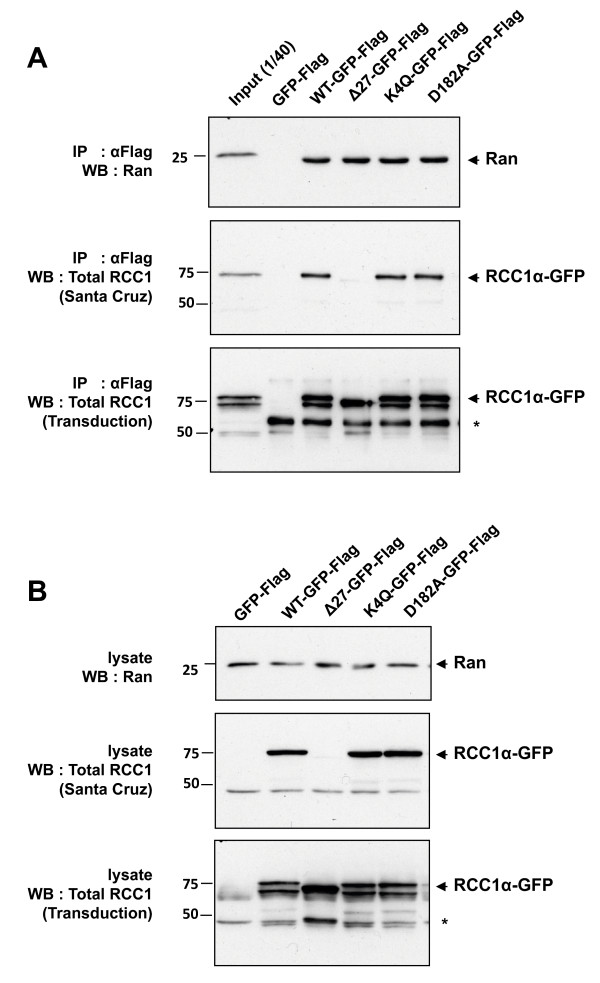
**Mutations of RCC1 that remove the N-terminal tail, block N-terminal methylation or inhibit GEF activity do not prevent its interaction with apoRan**. RCC1α wild-type (WT) and mutants (Δ27, K4Q and D182A) fused at the C-terminal with GFP-FLAG were expressed in U2OS cells (A) and immunoprecipitated with α-FLAG agarose beads (B). RCC1α-GFP-FLAG proteins were analysed by Western blotting using antibodies from Santa Cruz Biotechnology (sc-1162; does not recognize Δ27RCC1) (middle panel) and Transduction Laboratories (R35420) (bottom panel). The Transduction antibody detects both full-length RCC1α-GFP-FLAG and a lower band that is not detected by the Santa Cruz antibody. The latter therefore probably represents a truncated form lacking the N-terminal tail of RCC1α. A prominent non-specific band reacting with the Transduction antibody is indicated by *. Endogenous Ran present in the cell lysates (A) or co-immunoprecipitated with RCC1α-GFP-FLAG proteins (B) was detected by a specific antibody (top panel).

We tested further the relationship between the α-N-terminal methylation of RCC1α and stabilising effect of Ran^T24N ^on the interaction of RCC1α with chromatin. We found that the co-expression of mCherry-Ran^WT ^or mCherry-Ran^T24N ^in cells did not affect the α-N-terminal methylation of RCC1α-GFP or endogenous RCC1 when compared to the co-expression of mCherry alone (Figure 4). Therefore, Ran^T24N ^does not stabilise the interaction of RCC1α with chromatin by inducing the α-N-terminal methylation of RCC1α. Conversely, immunoprecipitation of RCC1α-GFP and RCC1α^K4Q^-GFP in the presence of EDTA, which chelates Mg^2+ ^and releases nucleotides from Ran to form apoRan [27], showed that endogenous Ran formed a stable complex with both RCC1α-GFP and RCC1α^K4Q^-GFP (Figure 5). Furthermore, removal of the N-terminal tail did not affect the association of apoRan with RCC1α under these conditions, showing that the methylated N-terminal tail is not required for binding to apoRan. These data indicate that the α-N-terminal methylation of RCC1α does not affect its association with apoRan. Thus, the α-N-terminal methylation of RCC1α and the association of the protein with Ran appear to be independent events.

### Inhibition of the GEF activity of RCC1α partially destabilises its interaction with chromatin but does not prevent a stabilising effect of Ran^T24N^

Mutation of aspartate 182 of RCC1α to alanine (D182A) strongly reduces k_cat _of its guanine nucleotide exchange activity towards Ran [12]. Aspartate 182 does not interact with Ran directly but rather forms an intramolecular hydrogen bond that stabilises the interaction of arginine 147 of RCC1α with the Ran P loop [11]. However, the D182A mutant of RCC1α has been found to disrupt mitosis [5,18] and does not rescue the effects of the mislocalisation of RCC1α through removal of the N-terminal tail [5]. These effects suggest that the D182A mutant has a dominant effect on the Ran system in cells and is not simply neutral.

In agreement with previous results using RCC1α tagged at the N-terminal with GFP [19] we found that interaction of RCC1α^D182A^-GFP with chromatin was more dynamic than the wild-type RCC1α-GFP, showing that this mutation decreased the stability of the interaction of RCC1α with chromatin (Figure 3). This suggests that the exchange activity of RCC1 promotes its interaction with chromatin. Nevertheless, we found that co-expression of mCherry-Ran^T24N ^stabilised the interaction of RCC1α^D182A^-GFP with chromatin in live cells. Consistent with this observation, RCC1α^D182A ^-GFP tagged with a FLAG epitope (RCC1α^D182A^-GFP-FLAG), like wild-type RCC1α-GFP-FLAG and RCC1α^K4Q^-GFP-FLAG, precipitated Ran from cells under Mg^2+^-chelated conditions, i.e. when a stable complex between RCC1α and apoRan is formed (Figure 5). Therefore, we show that D182A mutant of RCC1α still interacts with apoRan and this binary complex is sufficiently stable in the absence of guanine nucleotide binding for it to be co-precipitated. The stabilising effect of Ran^T24N ^on the interaction of RCC1α^D182A ^with chromatin indicates that the D182A mutation does not prevent the allosteric effect of Ran on the N-terminal tail of RCC1α.

## Discussion

In vitro, the stable association of nucleotide-free Ran with RCC1α produces a conformational change in the N-terminal tail that promotes binding to DNA and reduces affinity for core histones [13]. In cells, however, the interaction of RCC1 isoforms with chromatin is highly dynamic and is affected by post-translational modification of the tail. Our results confirm that this dynamic interaction is regulated by the association of Ran with RCC1α. A Ran mutant (Ran^T24N^) that associates stably with RCC1α in a binary complex strongly reduces the mobility of RCC1α on chromatin, and this effect requires the methylated N-terminal tail of RCC1α.

**Figure 6:**
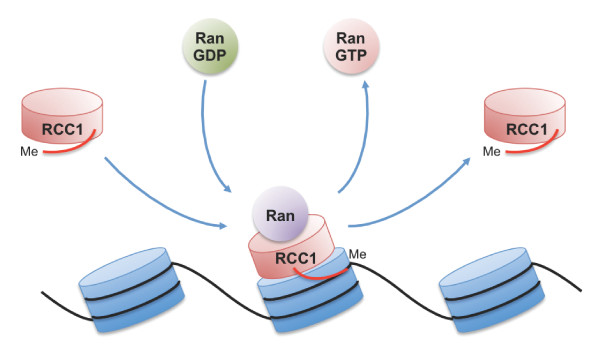
**Model of the dynamic interaction of RCC1 with chromatin**. RCC1 interacts weakly with a nucleosome through its core domain. Interaction of Ran-GDP releases GDP and leads to the transient formation of a binary complex between apoRan and RCC1 in which the methylated N-terminal tail undergoes a conformational change that allows it to interact with DNA and stabilise the association of RCC1. Binding of GTP to Ran causes dissociation of the binary complex and the release of RCC1 from the nucleosome.

Our results are consistent with a model in which apoRan (or Ran^T24N^) associates with the core domain of RCC1α and induces allosterically a conformational change in the N-terminal tail that stabilises interaction with chromatin. RCC1α associates weakly with chromatin through its core domain, possibly through interactions with core histones [13]. The interaction of nucleotide-bound Ran with RCC1α releases the nucleotide from Ran, forming a transient binary complex in which a conformational change in RCC1α exposes its N-terminal domain and stabilises its interaction with chromatin (Figure 6), possibly through direct interaction with DNA, although this remains to be confirmed in vivo. Other isoforms of RCC1 differ in the length of their N-terminal tails and in the turnover rate of their dynamic interactions with chromatin in cells [21], but it is likely that all isoforms interact with chromatin through a similar mechanism, albeit with differing affinities depending on the composition of the tail.

Our results with the D182A mutant of RCC1α, which inhibits guanine nucleotide exchange activity, are consistent with a model in which the dynamic interaction of RCC1α with chromatin is linked to its interaction with Ran. Previous experiments by Azuma and colleagues [12] using purified proteins have shown that the D182A mutant of RCC1α forms a binary complex with apoRan at a reduced rate compared to wild-type RCC1α, and the apoRan-RCC1α^D182A ^complex dissociates slowly even in the absence of free guanine nucleotide whereas the wild-type binary complex is stable. However, addition of guanine nucleotide causes rapid dissociation of both the RCC1α wild-type and D182A binary complexes [12]. Therefore the T24N mutation of Ran (which prevents nucleotide binding) can be envisaged to stabilise even the interaction with RCC1α^D182A ^to an extent and thereby promote the chromatin-binding conformation of this binary complex in cells. Nevertheless, the combination of reduced association rate of apoRan with RCC1α^D182A ^and likely increased dissociation rate of apoRan from RCC1α^D182A ^results in the interaction of RCC1α^D182A ^with chromatin being more dynamic than wild-type RCC1α even when Ran^T24N ^is present.

In agreement with the partial stabilising effect of Ran^T24N ^on the interaction of RCC1α^D182A ^with chromatin, we have also observed a complex formed between RCC1α^D182A ^and Ran under Mg^2+^-chelating conditions, which promote the nucleotide-free form of Ran (apoRan). This suggests that the apoRan-RCC1α^D182A ^binary complex might be more stable in cells than was predicted. Indeed, the stability of the wild-type apoRan-RCC1α binary complex when it interacts with chromatin is not yet known. A recent study of the interaction of RCC1α with nucleosomes in solution has suggested the interesting possibility that Ran could interact with RCC1α on chromatin in a different conformation from the crystallised binary complex [28], although the formation of such a distinct complex under cellular conditions remains to be confirmed. Whatever its conformation, the assembly of a complex between Ran and RCC1α^D182A ^that does not result in the efficient loading of Ran with GTP provides an explanation for the inhibitory effect of this mutant on mitosis [5,18].

The precise relationship between the dynamics of the interaction of RCC1α with chromatin and its guanine nucleotide exchange activity is, however, not yet certain. In one model, they are tightly coupled: binding of nucleotide to apoRan causes Ran-GTP (or Ran-GDP) to dissociate from RCC1α, then RCC1α is released from chromatin and the N-terminal tail folds back against the core domain (Figure 6). Although RCC1α can catalyse the reaction equally well from GDP to GTP and vice versa, the presence of accessory factors and the higher concentration of GTP than GDP in cells results in net loading of Ran with GTP. Alternatively, RCC1α could remain associated with chromatin for more than one guanine nucleotide exchange reaction if its dissociation from chromatin is slower than the release of Ran.

The conformational change in the N-terminal tail and/or its interaction with chromatin may be regulated by post-translational mechanisms as well as through the binding of Ran to RCC1 isoforms. Throughout the cell cycle, mono-, di- or tri-methylation of the α-amino group promotes the interaction with chromatin [18], whereas in mitosis, phosphorylation of serines 2 and 11 makes the interaction more dynamic [19,20]. We have found that α-N-terminal methylation of RCC1α is not affected by its stable association with Ran. We therefore favour the idea that post-translational modification of the N-terminal tail is relatively stable and is not tightly linked to the cycle of association and dissociation of RCC1 with chromatin, but rather alters the equilibrium of this interaction towards association with chromatin.

## Conclusions

Stabilisation of the dynamic interaction of RCC1α with chromatin by Ran requires the α-N-methylated N-terminal tail of RCC1α. This is consistent with the transient formation of the binary complex between RCC1 and Ran in which N-terminal tail of RCC1 undergoes a conformational change that allows it to interact more stably with chromatin. The coupling between the association of RCC1 with Ran and its interaction with chromatin provides an unusual mechanism to localise the generation of Ran-GTP.

## Methods

### Tissue culture

Human HeLa and U2OS cells obtained from Cancer Research UK London Institute were cultured in Dulbecco's Modified Eagle Medium (DMEM) (Invitrogen) supplemented with 10% Foetal Bovine Serum (Biosera), 50 units/ml Penicillin G (Invitrogen), 50 μg/ml Streptomycin (Invitrogen) and 2 mM L-glutamine (Invitrogen). Cells were grown at 37°C in 5% CO_2 _incubator. For FRAP experiments, cells were transfected with plasmids encoding GFP fusion constructs using Fugene HD transfection reagent following manufacturer's protocol. 24 hours post-transfection, media were replaced with phenol red-free DMEM (Invitrogen) and subject to imaging.

### Generation of GFP fusion constructs

All of the RCC1 constructs used in this study are derived from the major α isoform of human RCC1 [21]. To generate the GFP fusion RCC1α constructs, RCC1α N-terminal region (amino acids 1-27) was amplified and inserted into EcoRI and SalI sites of pEGFP-N1 (Clonetech) to generate the C-terminally GFP tagged vector expressing N-terminal tail of RCC1 α. Site directed mutagenesis was also used to introduce SV40 nuclear localization signal (NLS) into PstI site of both pEGFP-N1 vectors. The full-length sequence of RCC1 downstream of codon 27 (RCC1Δ27) was amplified and inserted into KpnI and BamHI sites or KpnI and AgeI sites of pEGFP-C3 and pEGFP-N1 vectors to generate GFP-Δ27RCC1 and Δ27RCC1-GFP fusion constructs, respectively. Following, site-directed mutagenesis was performed to introduce SV40 NLS into Pst I site of both vectors to generate GFP-SV40 NLS-Δ27 RCC1 (GFP-SVRCC1) and SV40 NLS-Δ27RCC1-GFP (SVRCC1-GFP), respectively.

Ran wild-type and T24N mutant cDNA were amplified from GFP-Ran^WT ^and GFP-Ran^T24N ^[26] and inserted into EcoRI and BamHI sites of an mCherry vector (a kind gift of Professor Angus Lamond, University of Dundee) to generate mCherry fusion constructs. The FLAG tag was inserted before the stop codon of GFP in pEGFP-N1 by site-directed mutagenesis. Sequences of the constructs made were verified by direct sequencing to ensure that no mutations were introduced.

### Fluorescent recovery after photobleaching (FRAP)

U2OS and HeLa cells were cultured in Phenol-red free DMEM (Invitrogen) on glass bottom dishes (35 × 22 mm) (Intracel). All constructs were fused with GFP at either C-terminus or N-terminus and expressed in Hela cells or U2OS cells where indicated. Fluorescence recovery after photobleaching (FRAP) experiments were performed on a DeltaVision Spectris microscopy workstation based on an Olympus IX70 inverted widefield deconvolution microsope equipped with QLM laser module. Live cells expressing GFP fusion and/or mCherry fusion constructs where indicated were scanned 3 times and GFP was photobleached using 488 nm laser at a defined spot on chromatin. Following, single images (512 × 512 pixels) were captured with the exposure time of 100 ms for 50 time points to monitor the FRAP half-time (t_1/2_). Data were processed with softWoRx software (Applied Precision).

### Western blotting

Proteins were resolved on SDS-PAGE gels and transferred onto nitrocellulose membrane (Amersham). Membranes were incubated in 5% dried skimmed milk/PBS-Tween (0.1%) for an hour and then with indicated primary antibodies diluted in 5% milk/PBS-Tween overnight at 4°C. Membranes were washed three times in PBS-Tween. Membranes were incubated with secondary antibodies diluted in 5% milk/PBS-Tween for an hour at room temperature and then washed three times in PBS-Tween.

### Antibodies

Goat anti-RCC1 polyclonal antibody (used at 1:1000 dilution) was from Santa Cruz Biotechnology (C-20: sc-1162). This antibody is described as having been raised against a C-terminal peptide, although we find that it does not recognize RCC1α lacking the first 27 residues (Δ27RCC1) (Additional file 3 Figure S3). Mouse anti-RCC1 monoclonal antibody from Transduction Laboratories (R35420). Mouse anti-FLAG monoclonal antibody (used at 1:8000 dilutions) and rabbit anti-actin polyclonal antibody (used at 1:5000 dilution) were from Sigma. Rabbit anti-α-dimethylated RCC1 polyclonal antibody (used at 1:1000 dilutions) was a kind gift of Dr. Ian Macara (University of Virginia). Goat anti-Ran (C-20: sc-1156) polyclonal antibody (used at 1:1000 dilution) was from Santa Cruz Biotechonology.

### FLAG Immunoprecipitation (IP)

Asynchronous U2OS cells were co-transfected with various RCC1 GFP-FLAG constructs and either mCherry empty vector or mCherry-Ran^WT ^fusion construct using Fugene HD transfection reagent (Roche) following manufacturer's protocol. 24 hours after transfection, cells were lysed in IP buffer (50 mM Tris pH 8.0, 2 mM EDTA pH 8.0, 150 mM sodium chloride, 50 mM sodium fluoride, 5 mM β-glycerophosphate, 1 mM sodium orthovanadate, 1% Triton X-100) supplemented with protease inhibitors and 1 mM okadaic acid. Lysates were left on ice for 20 minutes and spun down at 4°C for 20 minutes. FLAG beads (Sigma) were pre-washed 3 times in IP buffer. 1 mg lysates were then added to washed beads and incubated on wheel at 4°C for 90 minutes. Beads were washed with IP buffer 3 times. The immune complex was eluted with SDS loading buffer containing 5% β-mercaptoethanol. Samples were run on SDS-PAGE gel and blotted with indicated antibodies.

### Statistical analysis

Differences in mean of FRAP half-time (t_1/2_) was tested using Student's *t*-test (to compare means of 2 samples) and ANOVA test (to compare means of more than 2 samples) using statistics software (SPSS Inc.).

## Authors' contributions

EH generated and analysed the data. FEH made GFP-fusion constructs and provided preliminary data. HSS provided supporting data and participated in the design of the study. PRC conceived of the study and participated in its design. EH wrote the manuscript together with PRC. All authors read and approved the final manuscript.

## Supplementary Material

Additional file 1 **Figure S1**. Fluorescence recovery after photobleaching (FRAP) of RCC1α and mutants fused to GFP at the N-terminus or the C-terminus. Click here for file

Additional file 2 **Figure S2**. Fluorescence recovery after photobleaching (FRAP) showing stabilisation of the interaction of GFP-RCC1α (N-terminal GFP) with chromatin by Ran^T24N^. Click here for file

Additional file 3 **Figure S3**. Deletion of the N-terminal tail and mutation of lysine 4 (K4Q) abolishes methylation of RCC1α. Western blot showing the α-N-dimethylation of RCC1α, Δ27RCC1, RCC1α^K4Q^, RCC1α^D182A ^and RCC1α^S11A^. Click here for file
