# Supplementary material for: The methylated N-terminal tail of RCC1 is required for stabilisation of its interaction with chromatin by Ran in live cells
Source: BMC Cell Biol. 2010 Jun 21;11:43. doi: 10.1186/1471-2121-11-43 (PMC2898669; doi:10.1186/1471-2121-11-43)
Supplement: Additional file 1 — Figure S1. Fluorescence recovery after photobleaching (FRAP) of RCC1α and mutants fused to GFP at the N-terminus or the C-terminus. [file 1471-2121-11-43-S1.PDF]

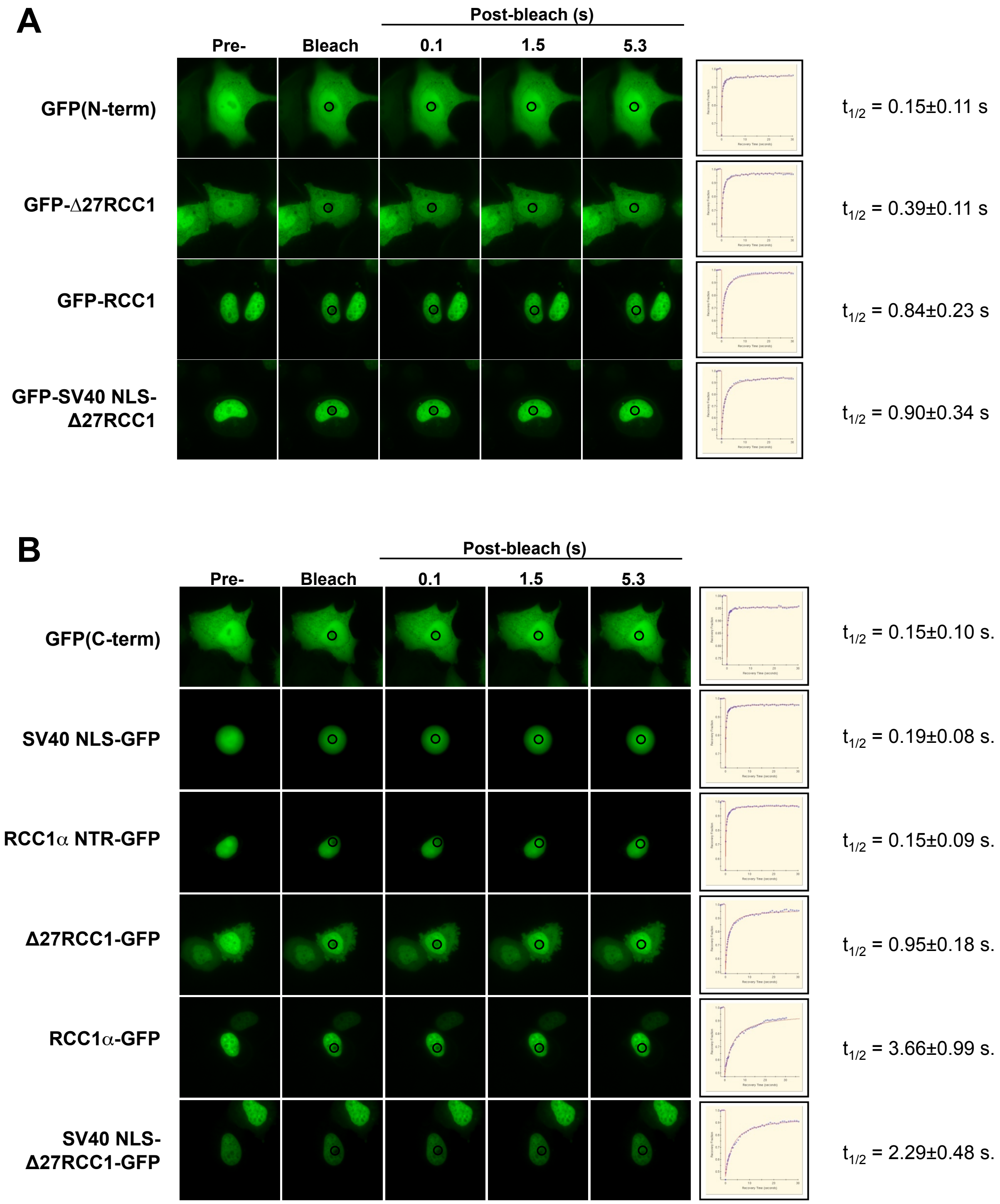

Additional file 1: Figure

Fluorescence recovery after photobleaching (FRAP) of RCC1α and mutants fused to GFP at the N-terminus (A) or the C-terminus (B). Images of a cell during the timecourse are shown (left). Circles indicate the region of photobleaching. Output of data (right) with FRAP half-time ($t_{1/2}$) +/- SD (s).
