# Supplementary material for: The methylated N-terminal tail of RCC1 is required for stabilisation of its interaction with chromatin by Ran in live cells
Source: BMC Cell Biol. 2010 Jun 21;11:43. doi: 10.1186/1471-2121-11-43 (PMC2898669; doi:10.1186/1471-2121-11-43)
Supplement: Additional file 2 — Figure S2. Fluorescence recovery after photobleaching (FRAP) showing stabilisation of the interaction of GFP-RCC1α (N-terminal GFP) with chromatin by RanT24N. [file 1471-2121-11-43-S2.PDF]

A

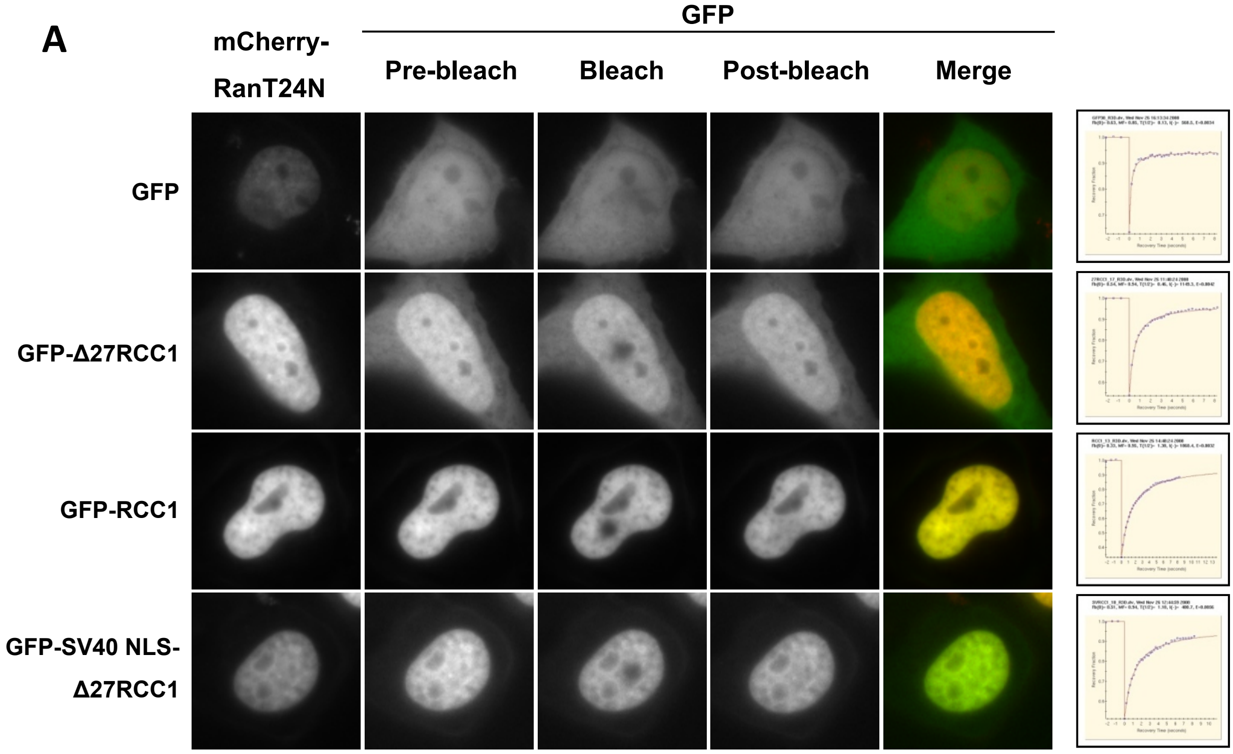

B

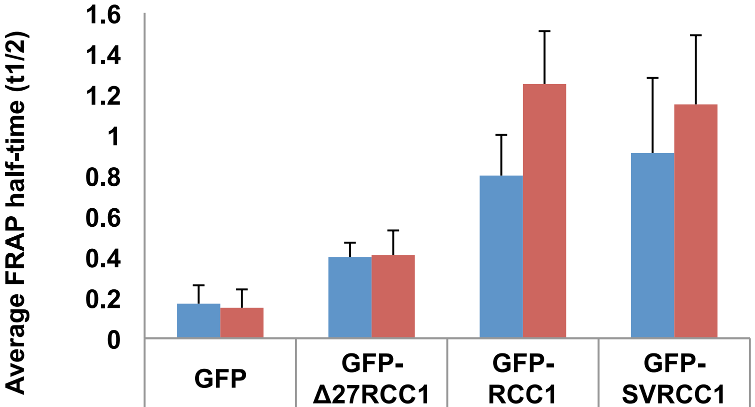

	GFP	GFP-Δ27RCC1	GFP-RCC1	GFP-SVRCC1
without mCherry-RanT24N	0.17	0.4	0.8	0.91
with mCherry-RanT24N	0.15	0.41	1.25	1.15

Additional file 2: Figure

Stabilisation of the interaction of GFP-RCC1α with chromatin by Ran^{T24N}. (A) Fluorescence recovery after photobleaching (FRAP) of RCC1α and mutants with GFP fused at the N-terminus co-expressed with $mCherryRan^{T24N}$. (B) Plot of FRAP data showing $t_{1/2}$ for GFP and fusions for cells with or without co-expressed mCherry-Ran^{T24N}.
