# Supplementary material for: The methylated N-terminal tail of RCC1 is required for stabilisation of its interaction with chromatin by Ran in live cells
Source: BMC Cell Biol. 2010 Jun 21;11:43. doi: 10.1186/1471-2121-11-43 (PMC2898669; doi:10.1186/1471-2121-11-43)
Supplement: Additional file 3 — Figure S3. Deletion of the N-terminal tail and mutation of lysine 4 (K4Q) abolishes methylation of RCC1α. Western blot showing the α-N-dimethylation of RCC1α, Δ27RCC1, RCC1αK4Q, RCC1αD182A and RCC1αS11A. [file 1471-2121-11-43-S3.PDF]

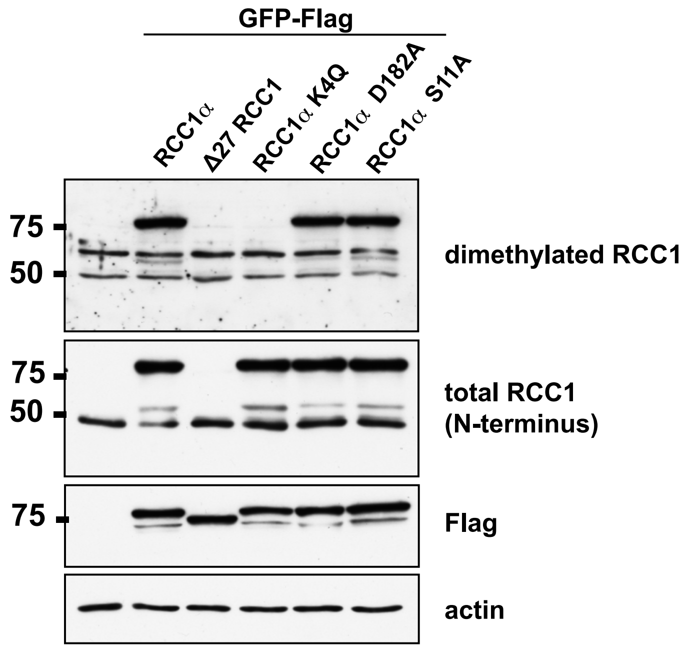

Additional file 3: Figure

Deletion of the N-terminal tail and mutation of lysine 4 (K4Q) abolishes methylation of RCC1α. RCC1α-GFP-Flag and mutants were expressed in U2OS cells. Cell lysates were blotted using antibodies recognising dimethylated RCC1, RCC1 (N-terminus), Flag epitope or actin as a loading control. The RCC1 antibody (sc-1162, Santa Cruz Biotechnology), which is described as being raised against a C-terminal peptide, nevertheless did not recognise RCC1 core domain (Δ27RCC1).
